# Mesothelin/Mucin 16 Signaling in Activated Portal Fibroblasts Drives the Development of Cholestatic Fibrosis and Hepatocellular Carcinoma in Aged Female Multidrug Resistance Protein 2 Knockout Mice

**DOI:** 10.1016/j.jcmgh.2026.101785

**Published:** 2026-04-11

**Authors:** Sadatsugu Sakane, Takahiro Nishio, Hiroaki Fuji, Se Yong Park, Kei Ishizuka, Charlene Miciano, Yusuke Kimura, Mojgan Hosseini, Karin Diggle, Vivian Zhang, Wonseok Lee, Hyun Young Kim, Xiao Liu, Allen Wang, David A. Brenner, Tatiana Kisseleva

**Affiliations:** 1 Department of Medicine, University of California, San Diego, La Jolla, California; 2 Department of Surgery, University of California, San Diego, La Jolla, California; 3 Department of Gastroenterology and Hepatology, The University of Osaka Graduate School of Medicine, Suita, Japan; 4 Department of Surgery, Graduate School of Medicine, Kyoto University, Kyoto, Japan; 5 Department of Gastroenterology, Juntendo University School of Medicine, Tokyo, Japan; 6 Department of Cellular and Molecular Medicine, Center for Epigenomics, University of California San Diego, La Jolla, California; 7 Department of Pathology, University of California, San Diego, La Jolla, California; 8 College of Pharmacy, Gachon University, Incheon, Republic of Korea; 9 College of Pharmacy, Dankook University, Cheonan, Chungnam, Republic of Korea; 10 Sanford Burnham Prebys Medical Discovery Institute, La Jolla, California; 11 Sanford Stem Cell Institute, University of California, San Diego, La Jolla, California

**Keywords:** Activated Portal Fibroblasts, Cholestatic Liver Fibrosis, Hepatocyte Regeneration, Hepatocellular Carcinoma

## Abstract

**Background & Aims:**

The contribution of activated hepatic stellate cells (aHSCs) to cholestatic fibrosis and cancer is well-documented, but the role of portal fibroblasts (PFs), and especially mesothelin (Msln)-mucin 16 (Muc16)- Thy-1 cell surface antigen (Thy-1) signaling in activated portal fibroblasts (aPFs), is unknown.

**Methods:**

The role of aPFs/mesenchymal cells in the pathogenesis of cholestatic fibrosis and hepatocellular carcinoma (HCC) was studied in aged (16 months old) multidrug resistance protein 2 knockout (Mdr2^−/−^) mice, which mimic primary biliary cholangitis with biliary fibrosis.

**Results:**

Aged female Mdr2^−/−^ mice were more susceptible to cholestatic fibrosis and inflammation and developed 4-fold more adenomas and GPC3^+^SOX9^+^AFP^+^ HCC than age-matched male littermates. Deletion of Msln or Muc16 ameliorated cholestatic fibrosis, inflammation and HCC in Mdr2^−/−^Msln^−/−^ and Mdr2^−/−^Muc16^−/−^ mice, whereas Mdr2^−/−^ and Mdr2^−/−^Thy-1^−/−^ mice exhibited similar phenotypes and developed severe fibrosis and HCC. Aged Mdr2^−/−^Msln^−/−^ and Mdr2^−/−^Muc16^−/−^ mice developed fewer HCCs and of smaller sizes. Ductular proliferation and hepatocyte and cholangiocyte senescence were suppressed in Mdr2^−/−^Msln^−/−^ and Mdr2^−/−^Muc16^−/−^ mice, whereas hepatocyte regeneration was markedly improved. Msln- and Muc16-deficient aPFs exhibited a less fibrogenic and inflammatory phenotype, and downregulated expression of Col1a2, Col3a1, Tgfβ1, MMP3, Cxcl9, Clcl7, Lgals1, and MMP2/3. The lack of MMP3 in Msln^−/−^ aPFs was linked to increased hepatocyte proliferation. Based on in vitro studies, MMP3-mediated shedding of hepatic HGFR (c-Met) was identified as one of the mechanisms by which aPFs suppress HGF-c-Met-induced phosphorylation of AKT, ERK, p38, resulting in proliferation of primary human hepatocytes. In turn, proliferation of MMP3-stimulated human hepatocytes was restored in the presence of MMP3 inhibitor.

**Conclusions:**

These findings demonstrate that aPFs mediate the crosstalk between cholangiocytes and hepatocytes, regulate hepatocyte functions, and that Msln-Muc16 signaling in aPFs is pathogenic for cholestatic fibrosis and HCC. Msln and Muc16 may become novel targets for anti-fibrotic therapy and patients with HCC and sclerosis cholangitis.

**Summary**

Mesothelin/mucin 16 signaling in activated portal fibroblasts promotes cholestatic fibrosis and hepatocellular carcinoma in aged female multidrug resistance protein 2^−/−^ mice. These findings identify a stromal signaling pathway linking fibrosis progression to liver tumorigenesis.

**What You Need to Know**

**Background**

The contribution of activated portal fibroblasts to cholestasis-associated liver cancer is not well understood.

**Impact**

In aged female Mdr2−/− mice, Msln/Muc16 signaling in activated portal fibroblasts promoted cholestatic fibrosis and HCC. Genetic deletion of Msln or Muc16 ameliorated cholestatic fibrosis, inflammation, and tumorigenesis, and improved HGF/c-Met-mediated hepatocyte proliferation and regeneration.

**Future Directions**

Targeting Msln+Muc16+ portal fibroblasts may become a new strategy for cholestatic fibrosis and liver cancer.

Although hepatotoxic liver injury is the most common cause of hepatocellular carcinoma (HCC),^1^ cholestatic liver injury can also lead to the development of HCC or intrahepatic cholangiocarcinoma (ICC) in patients with primary sclerosing cholangitis (PSC) and primary biliary cirrhosis (PBC). The incidence of PSC and PBC are rising, ranging from 0 to 16.2 per 100,000 people for PSC and 1.9 to 40.2 per 100,000 people for PBC.^2^ HCC may originate from de-differentiated mature hepatocytes, hepatic progenitors undergoing maturation arrest, or transformed senescent hepatocytes.^3^ HCC is identified by expression of alpha-fetoprotein (AFP), Yes-associated protein 1 (YAP), SRY-box transcription factor 9 (Sox9), glypican-3 (GPC3), and phospho-(p)-signal transducer and activator of transcription 3 (STAT3).^3^ The development of liver fibrosis and activation of fibrogenic myofibroblasts, mainly composed by activated hepatic stellate cells (aHSCs) and activated portal fibroblasts (aPFs)/mesenchymal cells, facilitates HCC progression.^4^ Although the role of aHSCs is well-documented,^5^ the contribution of aPFs to cholestasis-induced HCC is not fully understood.

aPFs produce fibrous scars around portal and periductular areas.^6^ Under physiological conditions, PFs represent only ≈0.1% of the cells in the liver, but they proliferate, upregulate collagen Type I, and contribute to >70% of myofibroblasts at the onset of injury, whereas HSCs activate later in the course of injury.^7^ aPFs can be distinguished from HSCs by expression of GPI-anchored Thy-1 cell surface antigen (Thy1) and mesothelin (Msln), transmembrane glycoprotein mucin 16 (Muc16), and other markers, such as CD34, Gremlin, Fibulin2, and Col15a.^7^ Msln-Muc16-Thy-1 signaling regulates transforming growth factor beta (TGFβ) responses in aPFs, and Msln^−/−^ and Muc16^−/−^ mice are protected from cholestatic fibrosis due to suppression of Smad2/3-dependent aPF activation.^8^ In turn, Thy-1 blocks TGFβ-TGFβRI-Msln-Muc16 signaling. Therefore, Thy1^−/−^ mice are more susceptible to cholestatic fibrosis due to increased activation of aPFs/mesenchymal cells.^8^ Meanwhile, the role of Msln-Muc16-Thy1 signaling in chronic cholestatic injury-associated liver cancer has not been studied.^9^

The current study investigates the pathogenesis of cholestatic fibrosis and cancer in multidrug resistance protein 2 knockout (Mdr2^−/−^) mice, which lack phosphatidylcholine excretion into the bile.^10^ Twelve-week-old Mdr2^−/−^ mice developed cholangitis and biliary fibrosis.^9^ Consistent with previous reports, 16-month-old Mdr2^−/−^ mice developed liver cancer.^11^ Here we demonstrate that female Mdr2^−/−^ mice developed 4.5 times more tumors, adenomas, and HCC but not ICC, than male Mdr2^−/−^ littermates. To dissect the role of aPF/mesenchymal cells in activation of cholestasis-induced HCC, Mdr2^−/−^ mice were crossed with either Msln^−/−^ mice, Muc16^−/−^ mice, or Thy1^−/−^ mice. The development of liver fibrosis and HCC was strongly suppressed in aged female Msln^−/−^Mdr2^−/−^ and Muc16^−/−^Mdr2^−/−^ mice (but not in Thy-1^−/−^Mdr2^−/−^ mice) vs Mdr2^−/−^ mice. This effect was attributed to a defect in aPF activation, and as a result, reduced hepatic injury, fibrosis, and inflammation. We demonstrate that aPFs regulate cholestasis-induced injury of hepatocytes. Hepatocyte regeneration was significantly improved in the livers of Msln^−/−^Mdr2^−/−^ and Muc16^−/−^Mdr2^−/−^ mice due to downregulation of aPF-derived matrix metalloproteinase-3 (MMP3), which induces shedding of hepatocyte growth factor receptor (HGFR; c-Met) from cholestatic hepatocytes. Here, we demonstrate that similar mechanism regulates proliferation of human hepatocytes, suggesting that targeting the Msln-Muc16 axis in aPFs/mesenchymal cells can prevent the development of age-related cholestatic fibrosis and HCC.

## Results

### Female Mdr2^−/−^ Mice Develop Liver Cancer With Age

**Figure 1:**
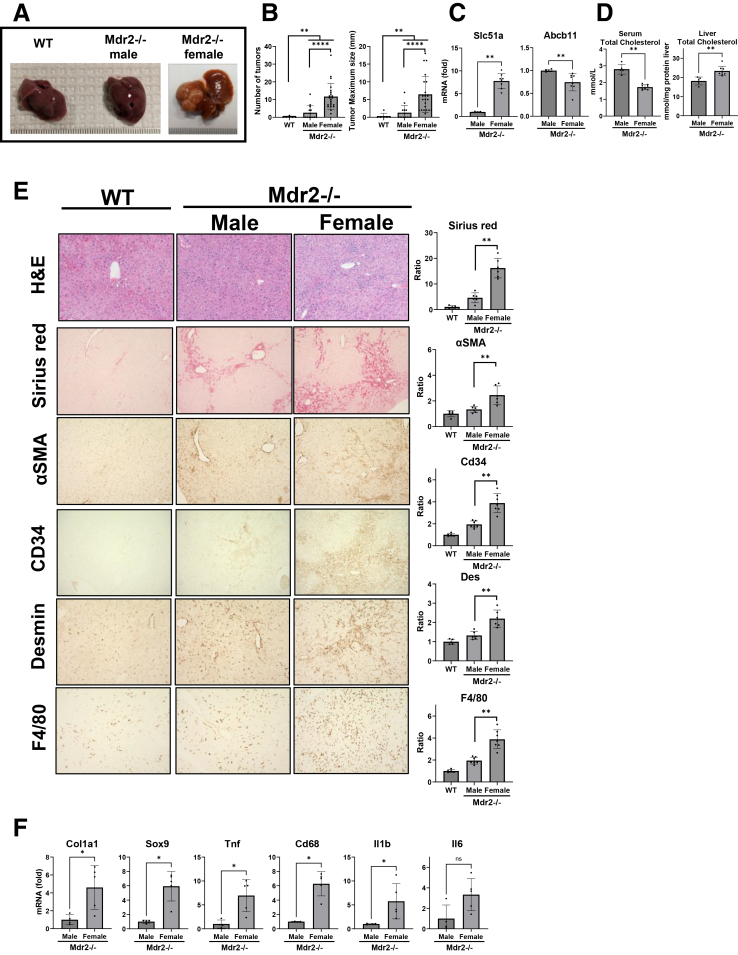
**Female Mdr2^−/−^ mice develop liver cancer with age.** Sixteen-month-old Mdr2^−/−^ mice (male and females, C57BL/6J, n ≥ 6). (*A*) Gross liver images. (*B*) The number and size of tumors were calculated. (*C*) Hepatic Slc51a and Abcb11 were measured. (*D*) Serum and hepatic levels of cholesterol were measured. (*E*) Livers were stained with H&E and Sirius red, anti-αSMA, anti-CD34, anti-Desmin, and anti-F4/80 Abs; representative micrographs are shown (10× objective). (*F*) Expression of fibrogenic and inflammation-related genes were analyzed by qRT-PCR. Data are presented as mean ± SD. The dot plot shows individual values. Comparisons between 2 groups were analyzed using the Mann-Whitney *U* test. ∗*P* < .05, ∗∗*P* < .01, ∗∗∗*P* < .001, ∗∗∗∗*P* < .0001.

Unlike wild-type (WT) mice, age-matched male and female Mdr2^−/−^ mice developed liver cancer with age (16 months old, C57BL6, n ≥ 6/sex). Gross liver examination revealed that 100% of aged female Mdr2^−/−^ mice developed cancer. Tumorigenesis was increased (>↑4-fold) in aged Mdr2^−/−^ females (vs aged Mdr2^−/−^ males), and females had more tumors of larger size (Figure 1*A* and *B*). The differences between female and male Mdr2^−/−^ mice have been previously noted, and these were attributed to the sexual dimorphism of bile acid (BA) synthesis and BA pool composition^12^ caused by estrogen-mediated upregulation of Slc51a (transports bile acids into the bloodstream) and downregulation of Abcb11 (transports bile acids into the bile canaliculi), and overall age-related hormonal changes in female Mdr2^−/−^ mice (Figure 1*C*).^13^^,^^14^ Moreover, despite low levels of serum cholesterol, hepatic levels of cholesterol are elevated in female Mdr2^−/−^ mice (Figure 1*D*). As a result, accumulation of hepatic cholesterol in aged female Mdr2^−/−^ mice exacerbated cholestatic injury, as shown by increased area of positive staining (≈↑20fold) for Sirius Red and alpha smooth muscle actin (αSMA), desmin^+^ HSCs and CD34^+^ aPFs, and F4/80^+^ myeloid cells, and upregulation (≈↑4-fold) of fibrogenic and proinflammatory genes (Col1a1, CD68, interleukin [IL]-1β, IL-6, and tumor necrosis factor [TNF]) (Figure 1*D* and *E*), and facilitated tumorigenesis.

### Genetic Deletion of Msln and Muc16 Suppresses Tumorigenesis in Aged Female Mdr2^−/−^ Mice

**Figure 2:**
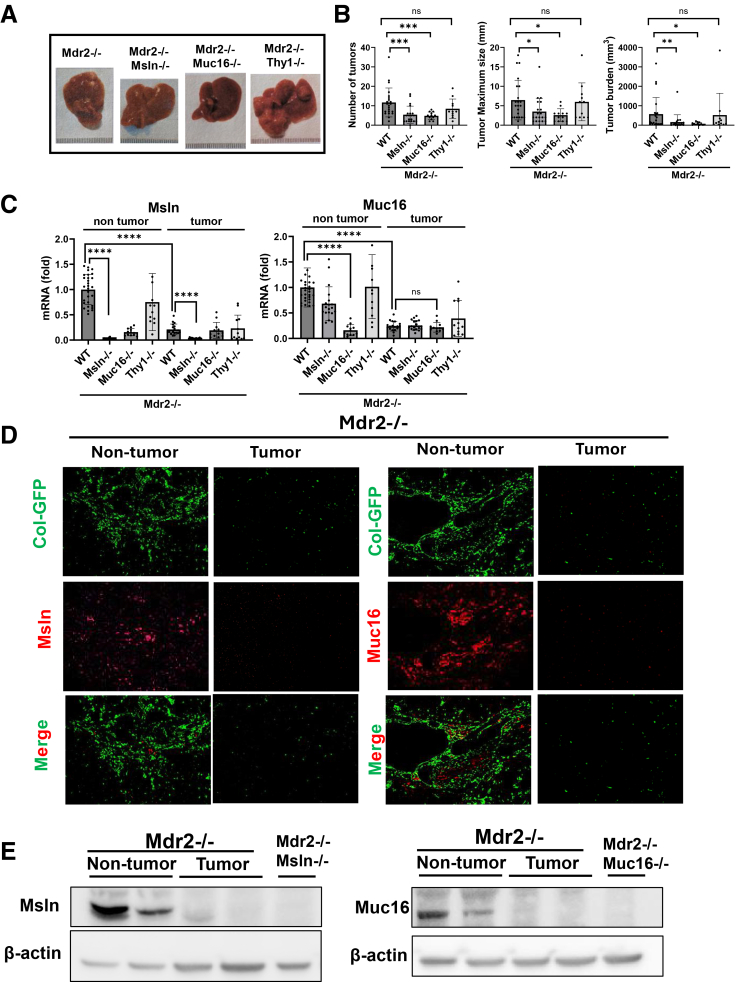
**Global deletion of Msln or Muc16 protects aged female Mdr2^−/−^ mice from HCC.** Sixteen-month-old female Mdr2^−/−^, Mdr2^−/−^Msln^−/−^, Mdr2^−/−^Muc16^−/−^, Mdr2^−/−^Thy1^−/−^ mice (C57BL/6J, n ≥ 12/sex/gorup). (*A*) Gross liver images. (*B*) Tumor number, size, and tumor burden were calculated. (*C*) Nontumor and tumor tissues were analyzed by qRT-PCR for expression of Msln and Muc16. (*D*) Livers of Mdr2^−/−^GFP aged mice were stained for markers Msln and Muc16. Micrographs were taken using 20× objective. (*E*) Expression of Msln and Muc16 in αSMA and β-actin was analyzed by Western blot. Data are presented as mean ± SD. The dot plot shows individual values. Comparisons between 2 groups were analyzed using the Mann-Whitney *U* test. ∗*P* < .05, ∗∗*P* < .01, ∗∗∗*P* < .001, ∗∗∗∗*P* < .0001.

The contribution of aPFs/mesenchymal cells to cholestatic fibrosis and cancer was further examined in aged female Mdr2^−/−^ mice. Mdr2^−/−^ mice were crossed with Msln^−/−^ mice, Muc16^−/−^ mice, or Thy1^−/−^ mice. The role of Msln-Muc-16-Thy1 signaling in aPFs/mesenchymal cells in the pathogenesis of cholestatic fibrosis and cancer was evaluated in aged female Mdr2^−/−^Msln^−/−^, Mdr2^−/−^Muc16^−/−^, Mdr2^−/−^Thy1^−/−^ , and Mdr2^−/−^ mice (n ≥ 12/sex/group). In comparison with Mdr2^−/−^ mice, deletion of Msln or Muc16 protected Mdr2^−/−^Msln^−/−^ and Mdr2^−/−^Muc16^−/−^ mice from tumorigenesis, as they developed fewer tumors of smaller size (Figure 2*A* and *B*). Tumor burden was significantly lower in aged female Mdr2^−/−^Msln^−/−^ and Mdr2^−/−^Muc16^−/−^ mice (vs Mdr2^−/−^ mice) (Figure 2*B*).

In turn, Mdr2^−/−^Thy1^−/−^ mice developed more tumors (≈↑2-fold increase vs Mdr2^−/−^ mice), although the tumor burden was not significantly changed between Mdr2^−/−^Thy1^−/−^ and Mdr2^−/−^ mice (Figure 2*B*). Our data suggest that Msln and Muc16 (but not Thy-1) are critical for activation of aPFs and pathogenic for the development of cholestatic fibrosis and cancer.

### Aged Female Mdr2^−/−^Msln^−/−^ and Mdr2^−/−^Muc16^−/−^ Mice Are Protected From HCC

In adult mice and humans, Msln and Muc16 are expressed by mesenchymal stem cells^15^ or cancer cells, such as pancreatic, ovarian cancer, or ICC.^16^^,^^17^ Since neither Msln nor Muc16 were expressed in tumors from Mdr2^−/−^ mice, as shown by quantitative reverse transcription polymerase chain reaction (qRT-PCR), immunostaining, and Western blot analysis (Figure 2C‒E), the tumor suppressive phenotype in Mdr2^−/−^Msln^−/−^ and Mdr2^−/−^Muc16^−/−^ mice was linked to reduced activation/proliferation of aPF/mesothelial cells.

**Figure 3:**
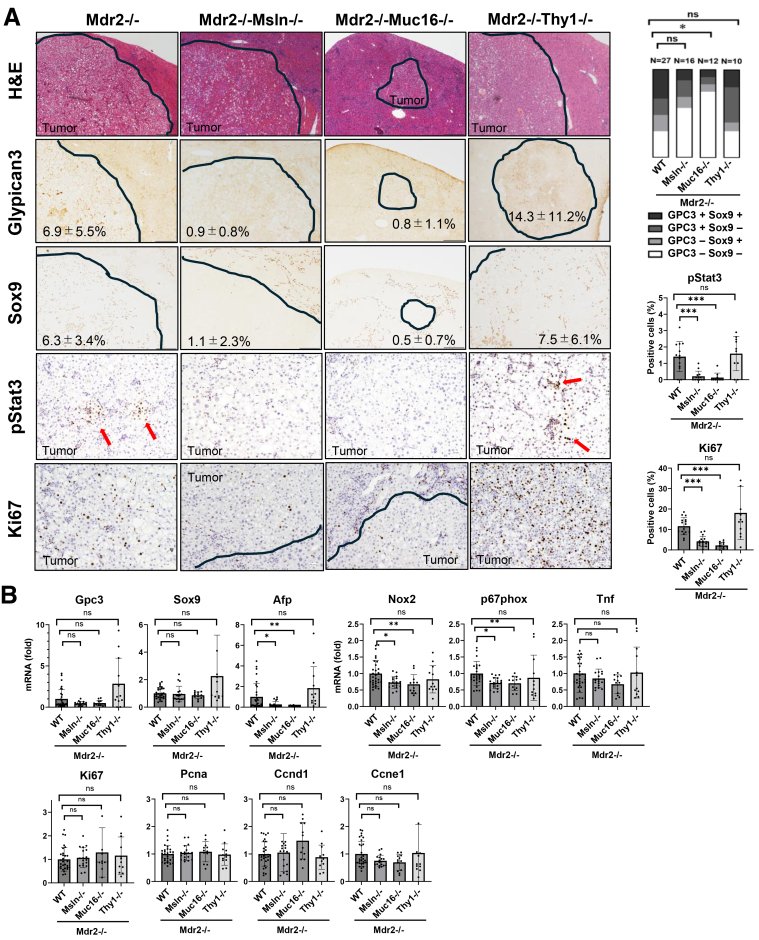
**Msln^−/−^ or Muc16^−/−^ tumors exhibit less malignant phenotype in aged female Mdr2^−/−^ mice.** (*A*) Livers were stained with H&E, anti-GPC3, anti-Sox9 (Sox9), anti-phospho-Stat3 (pStat3), and anti-Ki67 Abs. Tumors were analyzed, the number of positive tumors was calculated, representative micrographs are shown (10× objectives). (*B*) Tumors (<3 mm) were analyzed by qRT-PCR. Expression of HCC-associated, ROS, and proliferation-associated genes was analyzed by qRT-PCR in tumors of these mice. Data are presented as mean ± SD. Comparisons between 2 groups were analyzed using the Mann-Whitney *U* test. Categorical data were analyzed by Fisher's exact test. ∗*P* < .05, ∗∗*P* < .01, ∗∗∗*P* < .001, ∗∗∗∗*P* < .0001.

Liver tumors were further characterized by a pathologist in a double blinded manner (see Methods). Unlike hepatocellular adenoma (HCA), HCC is a malignant liver cancer characterized by expression of AFP, Sox9, and GPC3.^3^ Histopathology (hematoxylin and eosin [H&E]) and immunostaining for GPC3, Sox9, and phospho-(p)-Stat3 revealed that Mdr2-deficiency caused the development of hepatic adenomas and HCC in aged female Mdr2^−/−^ mice (but not ICC) (Figure 3*A*).^11^ Overall, the aged female Mdr2^−/−^ mice developed the largest number of tumors, identified as double-positive GPC3^+^Sox9^+^ and single-positive GPC3^+^ or Sox9^+^ HCC, and double-negative GPC3^-^Sox9^-^ HCA. In turn, the number of tumors (HCC + adenomas) were markedly reduced in aged female Mdr2^−/−^Msln^−/−^ (↓50%) and Mdr2^−/−^Muc16^−/−^ mice (↓50%) vs Mdr2^−/−^ mice, with the prevalence of HCA over HCC, as shown by the higher ratio (4:1) of GPC3^-^Sox9^-^ to GPC3^+^Sox9^+^ tumors (Figure 3*A*). The number of p-Stat3^+^ tumors was reduced in these mice. The expression of AFP, NADPH oxidase 2 (Nox2) and p67phox messenger RNA (mRNA) was significantly lower in Mdr2^−/−^Msln^−/−^ Mdr2^−/−^Muc16^−/−^ tumors (vs with Mdr2^−/−^ tumors) (Figure 3*B*). Although the number of GPC3^+^Sox9^+^ HCC was somewhat reduced in Mdr2^−/−^Thy1^−/−^ mice, livers of Mdr2^−/−^Thy1^−/−^ and Mdr2^−/−^ mice had similar tumor size and number and phospho-Stat3 expression, suggesting that deletion of Thy-1 does not significantly affect the development of cholestasis-induced cancer (Figures 3*A* and *B*).

### Cholestatic Fibrosis Is Suppressed in Aged Female Mdr2^−/−^Msln^−/−^ and Mdr2^−/−^Muc16^−/−^ Mice

**Figure 4:**
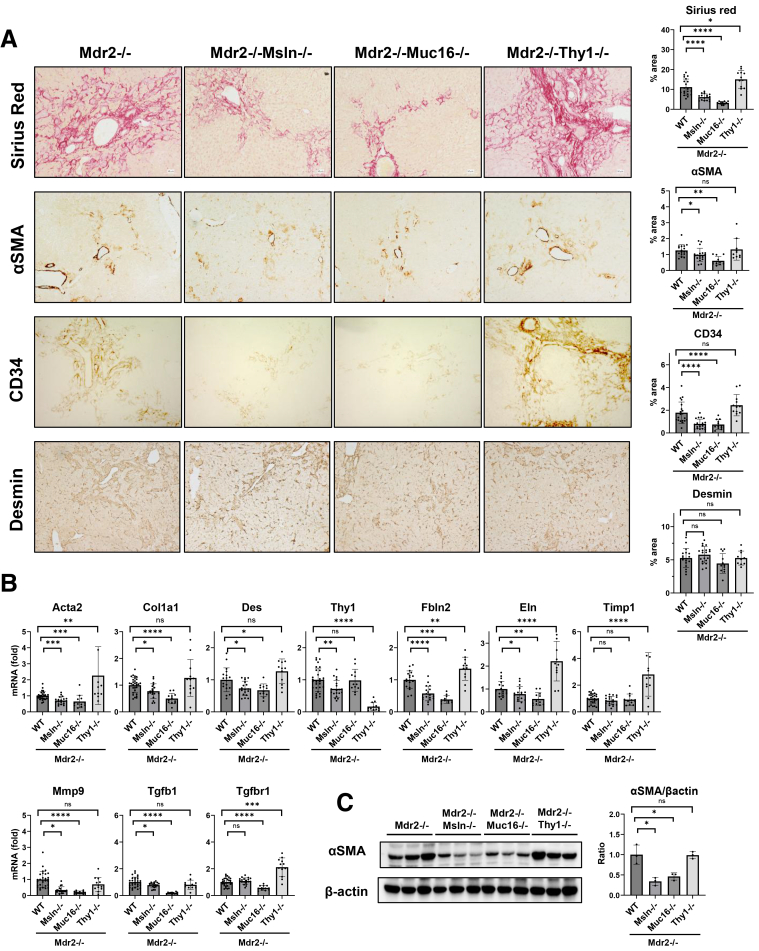
**Global deletion of Msln or Muc16 ameliorates liver fibrosis in aged female Mdr2^−/−^ mice.** (*A*) Livers were stained with Sirius Red, anti-αSMA, anti-CD34, and anti-Desmin Abs. Positive area was calculated as percent. Micrographs are taken using 20× objectives. (*B*) Livers were analyzed by qRT-PCR for expression of fibrogenic genes and aPF-specific genes. (*C*) Expression of αSMA and β-actin was analyzed by Western blot. Data are presented as mean ± SD. The dot plot shows individual values. Comparisons between 2 groups were analyzed using the Mann-Whitney *U* test. ∗*P* < .05, ∗∗*P* < .01, ∗∗∗*P* < .001, ∗∗∗∗*P* < .0001.

As expected,^9^ aged female Mdr2^−/−^Msln^−/−^ and Mdr2^−/−^Muc16^−/−^ mice developed less fibrosis, as shown by reduced area of staining for Sirius red, αSMA, and aPF-marker CD34, but not HSC-marker Desmin, in the livers of these mice compared with aged female Mdr2^−/−^ mice (Figure 4*A*). Expression of fibrogenic genes (Acta2, Col1a1, Mmp9, and TGFβ mRNA), and αSMA protein was reduced in Mdr2^−/−^Msln^−/−^ and Mdr2^−/−^Muc16^−/−^ mice (Figure 4*B* and *C*).

In comparison, expression of Acta2, Col1a1, Timp1, and TGFβRI mRNA and αSMA protein was increased in Mdr2^−/−^Thy1^−/−^ mice (vs Mdr2^−/−^ mice), whereas positive staining for Sirius red, αSMA, CD34, and Desmin was not significantly changed between these mice (Figure 4*B* and *C*), suggesting that Thy1 is dispensable for the development of cholestatic fibrosis in aged female mice.

### Ductular Reaction and Inflammation Are Reduced in Aged Female Mdr2^−/−^Msln^−/−^ and Mdr2^−/−^Muc16^−/−^ Mice

**Figure 5:**
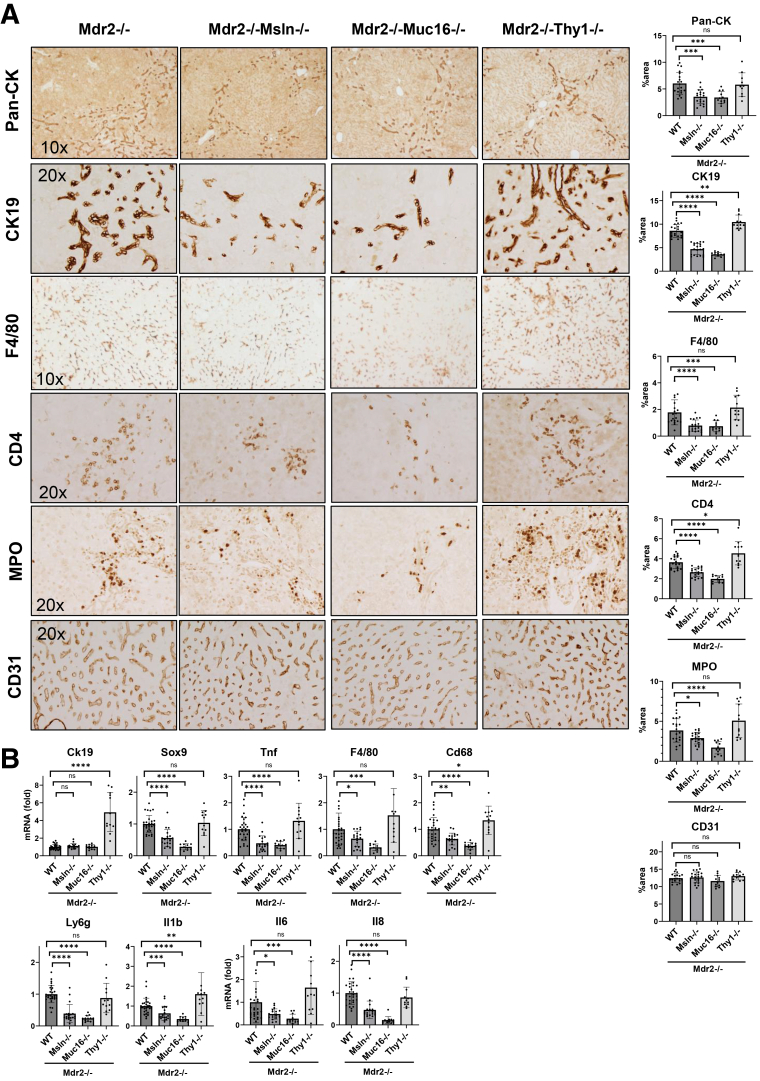
**Global deletion of Msln or Muc16 prevents ductular proliferation and inflammation in aged female Mdr2^−/−^ mice.** (*A*) Livers were stained with anti-Pan-CK and anti-F4/80, anti-CK19, anti-CD4, anti-MPO and anti-CD31. Positive area was calculated as percent. Micrographs were taken using 10× or 20× objectives. (*B*) Livers were analyzed by qRT-PCR for expression of epithelial and inflammatory markers. Data are presented as mean ± SD. The dot plot shows individual values. Comparisons between 2 groups were analyzed using the Mann-Whitney *U* test. ∗*P* < .05, ∗∗*P* < .01, ∗∗∗*P* < .001, ∗∗∗∗*P* < .0001.

Msln and Muc16 regulate ductular reaction in young Mdr2^−/−^ mice.^8^^,^^9^ Indeed, cholangiocyte-specific expression of pan-cytokeratin (Pan-CK) and Sox9 was decreased (≈↓50%) in the livers of aged female Mdr2^−/−^Msln^−/−^ and Mdr2^−/−^Muc16^−/−^ mice (vs Mdr2^−/−^ mice) (Figure 5*A* and *B*). The number of F4/80^+^ myeloid cells, CD4 T cells, and myeloperoxidase (MPO)^+^ neutrophils (but not endothelial cells), as well as expression of inflammatory genes (F4/80, CD68, Ly6G, IL1β, IL6, and IL8) was also reduced in the livers of aged female Mdr2^−/−^Msln^−/−^ and Mdr2^−/−^Muc16^−/−^ mice, suggesting that Msln-Muc16 signaling in aPFs/mesenchymal cells mediates a crosstalk between cholangiocytes and myeloid cells. In contrast, deletion of Thy1 minimally affected ductular proliferation and inflammation in Mdr2^−/−^Thy1^−/−^ mice vs Mdr2^−/−^ mice (Figure 5*A* and *B*).

### Senescence of Liver Parenchymal Cells Is Suppressed in Aged Female Mdr2^−/−^Msln^−/−^ and Mdr2^−/−^Muc16^−/−^ Mice

**Figure 6:**
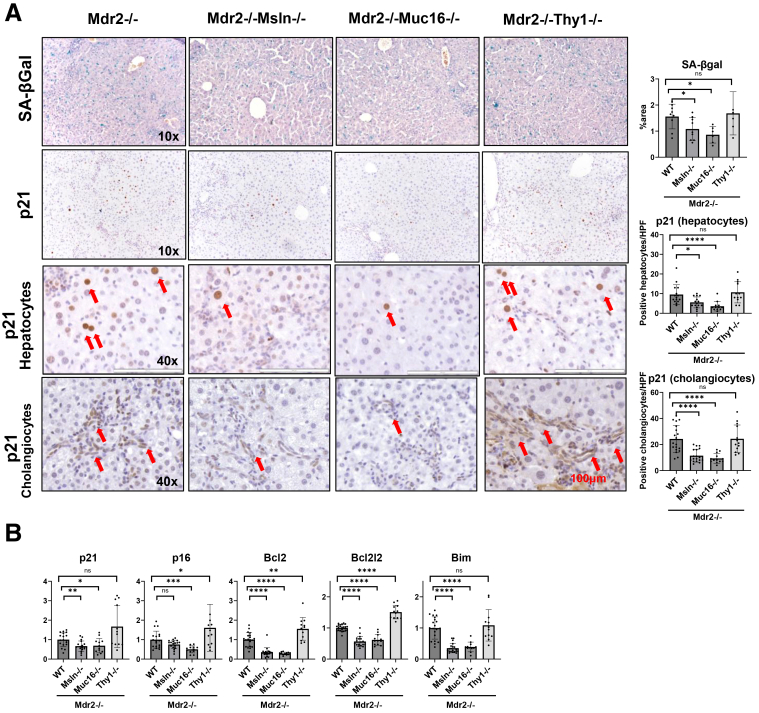
**Global deletion of Msln or Muc16 prevents hepatocyte and cholangiocyte senescence in aged female Mdr2^−/−^ mice.** (*A*) Livers were stained with SA-βGal and anti-p21 Ab. SA-βGal positive staining was calculated as percent. The number of positive senescent p21+ hepatocytes/cholangiocytes per high power field (HPF) was counted. Representative micrographs are taken using 10× and 40× objectives. (*B*) Livers were analyzed by qRT-PCR for expression of senescent markers. Data are presented as mean ± SD. The dot plot shows individual values. Comparisons between 2 groups were analyzed using the Mann-Whitney *U* test. ∗*P* < .05, ∗∗*P* < .01, ∗∗∗*P* < .001, ∗∗∗∗*P* < .0001.

Cholestasis-induced senescence of parenchymal cells is linked to the development of liver injury and inflammation.^18^ When expression of senescence-associated markers was examined in nontumor liver tissue, SA-βGal, p21, p16, Bcl-2, Bcl2l2, and Bim genes were downregulated in aged female Mdr2^−/−^Msln^−/−^ and Mdr2^−/−^Muc16^−/−^ mice compared with Mdr2^−/−^ mice (Figure 6*A* and *B*), indicating that blocking of Msln-Muc16 signaling in aPFs/mesenchymal cells prevents hepatocyte/cholangiocyte senescence. Moreover, hepatocyte senescence and survival was not significantly changed between Mdr2^−/−^Thy-1^−/−^ and Mdr2^−/−^ mice (Figure 6*A* and *B*).

### aPFs Do Not Serve as a Significant Source of Tumor-Associated Myofibroblasts in Aged Female Mdr2^−/−^ Mice

**Figure 7:**
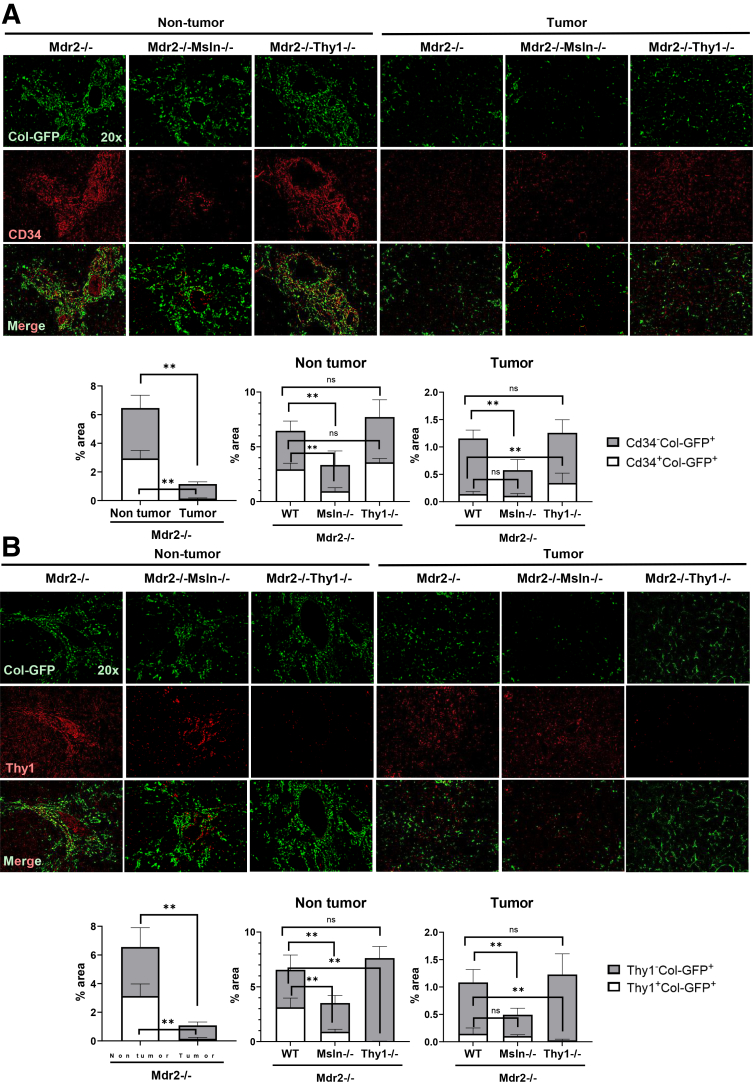
**Liver fibrosis is suppressed and hepatocyte proliferation is improved in aged female Mdr2^−/−^Msln^−/−^ mice.** Livers of Mdr2^−/−^GFP+, Mdr2^−/−^Msln^−/−^GFP+, and Mdr2^−/−^Thy1^−/−^GFP+ aged mice (female, C57BL/6, n ≥ 7/group) were stained for aPF-specific markers (*A*) CD34 and (*B*) Thy1; overlapping area was calculated in nontumor and tumor liver tissues as percent, representative micrographs (20× objective). Positive area was calculated as percent. Comparisons between 2 groups were analyzed using the Mann-Whitney *U* test. ∗*P* < .05, ∗∗*P* < .01.

Both fibrogenic (nontumor) and tumor-associated myofibroblasts (cancer-associated fibroblasts [CAFs]) were shown to promote HCC progression.^19^ Previous studies have suggested that Msln is a dominant regulator of Msln-Muc16 signaling-dependent activation and proliferation of aPFs.^8^ To investigate the role of aPFs in HCC growth, Col-GFP reporter mice, which upregulate GFP in real time in all Collagen-1α(I)-expressing cells,^20^ were crossed with Mdr2^−/−^, Mdr2^−/−^Msln^−/−^, or Mdr2^−/−^Thy1^−/−^ mice (Figure 7*A* and *B*). All hepatic myofibroblasts in these mice were visualized by expression of GFP. Livers were stained for aPF-marker CD34 and Thy1. Nontumor and tumor areas were analyzed for the presence of CD34^+^GFP^+^ or Thy1^+^GFP^+^aPFs, and CD34^-^Thy1^-^GFP^+^ aHSCs. In aged female Mdr2^−/−^GFP^+^ mice, GFP^+^ fibrogenic myofibroblasts were composed of 50% aHSCs and 50% aPFs. The ratio of fibrogenic to tumor-associated aHSCs was 3:1, whereas the ratio of fibrogenic to tumor-associated aPFs was ≈20:1, suggesting that aPFs do not serve as a significant source of CAFs (Figure 7*A* and *B*). Importantly, the number of tumor-associated aHSCs was much higher than aPFs in the livers of Mdr2^−/−^GFP^+^ mice, implying that tumor-associated aHSCs^19^ (but not aPFs) critically regulate the development of HCC. Next, the myofibroblast composition was compared in the livers of Mdr2^−/−^GFP^+^, Mdr2^−/−^Msln^−/−^GFP^+^, or Mdr2^−/−^Thy1^−/−^GFP^+^ mice.

### The Number of Tumor-Associated aPFs Is Reduced in Aged Female Mdr2^−/−^Msln^−/−^Col-GFP^+^ Mice

The proportion of tumor-associated aPFs vs fibrogenic aPFs was consistently lower across all genotypes (Figure 7*A* and *B*). The number of aPFs and, surprisingly, aHSCs was reduced (↓50%) within tumors of Mdr2^−/−^Msln^−/−^GFP^+^ mice. In turn, the number of tumor-associated CD34^+^GFP^+^ aPFs was increased (↑2-fold) in aged female Thy-1^−/−^Mdr2^−/−^GFP^+^ mice compared with Mdr2^−/−^GFP^+^ mice (Figure 7*A*), whereas the number of aHSCs or tumor burden was not significantly changed between these mice (Figure 7*B*). We suggest that due to the low abundance, tumor-associated aPFs might not significantly affect the progression of cholestasis-induced HCCs.

### Msln Signaling in Fibrogenic aPFs Regulates the Development of Cholestatic Fibrosis and HCC in Aged Female Mdr2^−/−^Msln^−/−^Col-GFP^+^ Mice

In nontumor area, the number of fibrogenic aPFs was markedly decreased (↓3-fold) in aged female Mdr2^−/−^Msln^−/−^GFP^+^ mice, whereas the number of Mdr2^−/−^Thy1^−/−^GFP^+^ aPFs was increased (↑3-fold) compared with Mdr2^−/−^GFP^+^ aPFs. Remarkably, the number of aHSCs remained unchanged between all groups of mice (Figure 7*A*). Because aPFs minimally contributed to tumor-associated CAFs in Mdr2^−/−^ mice (Figure 7*A* and *B*), the tumor suppressive phenotype of Mdr2^−/−^Msln^−/−^ and Mdr2^−/−^Muc16^−/−^ mice was attributed to impaired activation of fibrogenic aPF/mesenchymal cells. Therefore, nontumor liver tissue of aged female mice was examined further.

### Basal Level of Hepatocyte Regeneration Is Increased in Aged Mdr2^−/−^Msln^−/−^ and Mdr2^−/−^Muc16^−/−^ Mice

**Figure 8:**
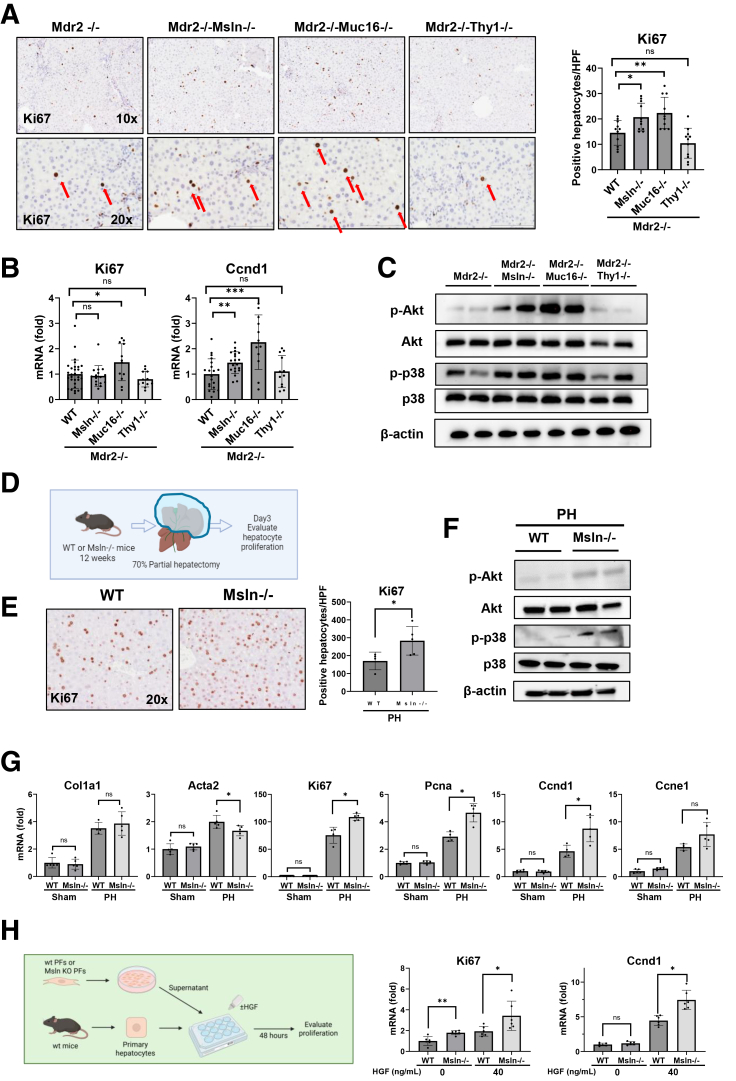
**Hepatocyte proliferation is improved by Msln^−/−^ PFs.** (*A*) Livers were stained with anti-Ki67 Abs. Positive area was calculated as percent. The number of positive hepatocytes per high power field (HPF) was counted (10× and 20× objectives). (*B*) Expression of proliferation markers was analyzed in these mice using qRT-PCR, or (*C*) Western blotting for phospho-Akt, Akt, phospho-p38, and p38. (*D‒G*) A 70% partial hepatectomy (PH) or sham operation was performed using 12-week-old WT or Msln^−/−^ mice (female, C57BL/6, n ≥ 4/group), and mice were sacrificed 3 days later. (*D*) Study design. (*E*) Livers were stained with anti-Ki67 Abs. Positive area was calculated as percent. The number of positive hepatocytes per high power field (HPF) was counted (20× objectives). (*F*) Expression of proliferation markers was analyzed in these mice using Western blotting for phospho-Akt, Akt, phospho-p38, and p38, or (*G*) qRT-PCR. (*H*) Primary hepatocytes were isolated from 8-week-old WT mice (male, C57BL/6) and seeded, and cultured in conditioned medium from WT aPFs or Msln^−/−^ aPFs ± mouse recombinant HGF (for 48 hours). Expression of proliferation markers was assessed by qRT-PCR. Data are presented as mean ± SD. The dot plot shows individual values. Comparisons between 2 groups were analyzed using the Mann-Whitney *U* test. ∗*P* < .05, ∗∗*P* < .01, ∗∗∗*P* < .001, ∗∗∗∗*P* < .0001.

We tested if the regenerative capacity of nontumor hepatocytes was improved in aged female Mdr2^−/−^Msln^−/−^ and Mdr2^−/−^Muc16^−/−^ mice. Indeed, hepatocyte proliferation (Ki67 and Ccnd1 expression) was increased in the livers of aged female Mdr2^−/−^Msln^−/−^ and Mdr2^−/−^Muc16^−/−^ mice (vs Mdr2^−/−^Thy1^−/−^ and Mdr2^−/−^ mice), as shown by upregulation of Ki67 and CyclinD1 (Figure 8*A* and *B*), and activation of phospho-Act1 and phospho-p38, which expression was linked to hepatocyte proliferation (vs Mdr2^−/−^Thy1^−/−^ mice and Mdr2^−/−^ mice) (Figure 8*C*). These results suggest that deletion of Msln or Muc16 in aPFs can improve hepatocyte regeneration in mice with cholestasis.

### Liver Regeneration Is Accelerated in Young Msln^−/−^ Mice

To test this hypothesis, liver regeneration was assessed in young (12 weeks old, n ≥ 4/group) WT and Msln^−/−^ mice. WT and Msln^−/−^ mice were subjected to partial (70%) hepatectomy, and livers were analyzed 3 days later (Figure 8*D*). Hepatocyte proliferation was markedly increased in Msln^−/−^ mice compared with WT mice, shown by upregulation of Ki67 (↑1.5-fold), phospho-Akt (↑3-fold), and phospho-38 (↑2-fold) in livers of Msln^−/−^ mice (Figure 8*E* and *F*), and increased expression of Ki67, Pcna, Ccnd1, and Ccne1 mRNA (Figure 8*G*).

### Soluble Factors Secreted by Msln^−/−^ aPFs Promote Hepatocyte Proliferation

To access the mechanism by which aPFs mediate hepatocyte proliferation, primary aPFs were sort-purified from cholestatic livers of bile duct ligation (BDL)-injured Col-GFP^+^ WT and Msln^−/−^Col-GFP^+^ mice, and analyzed by RNA sequencing (RNA-seq), or immortalized.^9^ Col-GFP^+^ WT and Msln^−/−^Col-GFP^+^ aPFs were cultured, and the supernatant was collected and used for in vitro stimulation of freshly isolated primary mouse WT hepatocytes.

When cultured in conditioned media from Msln-deficient WT aPFs (vs media from WT aPFs), in vitro proliferation of primary mouse WT hepatocytes was increased (↑1.7-fold). Remarkably, proliferation of WT hepatocytes was synergistically increased by addition of HGF to conditioned media from Msln-deficient WT aPFs. Our data indicate that WT aPFs suppress physiological growth and regeneration of hepatocytes by secreting soluble chemokines/cytokines that inhibit hepatocyte proliferation but promote inflammation and hepatocyte senescence (Figure 8*H*).

### Msln^−/−^ aPFs Downregulate Profibrogenic and Inflammatory Genes

**Figure 9:**
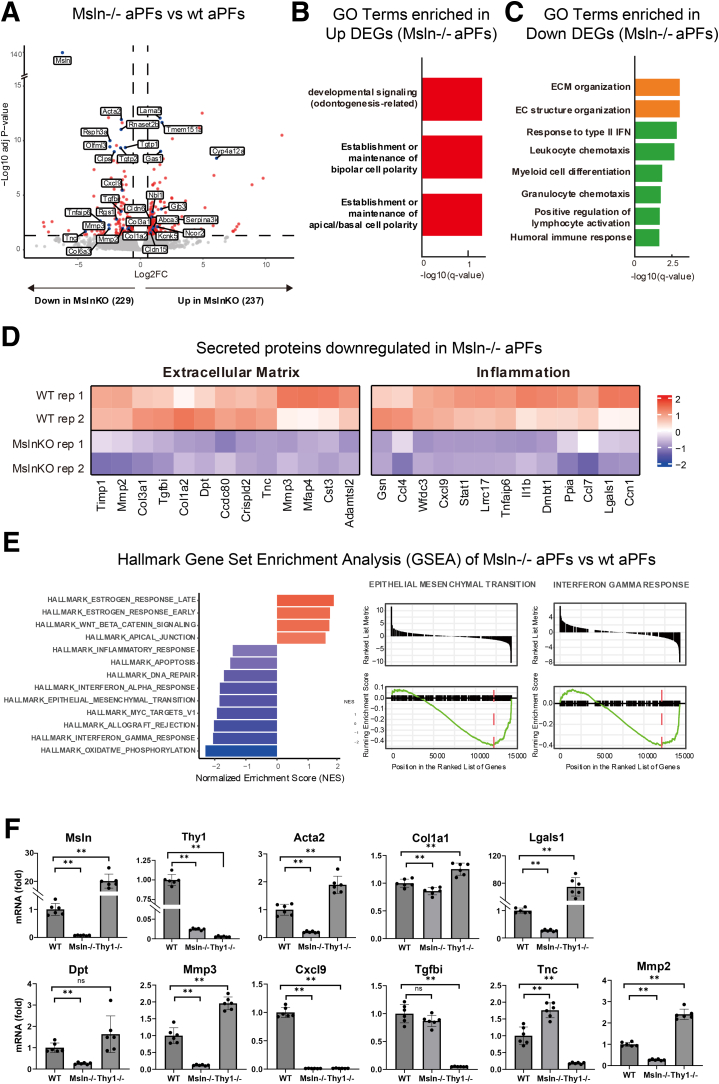
**Fibrogenic and inflammatory responses are suppressed in Mdr2^−/−^Msln^−/−^ aPFs.** (*A*) Volcano plot showing DEGs in Msln^−/−^ vs WT aPFs (adjusted *P* < .05; |log_2_FC| > .58). (*B*) GOBP overrepresentation analysis of genes upregulated in Msln^−/−^ aPFs highlights enrichment of developmental signaling (odontogenesis-related) and apical/basal polarity programs. (*C*) GOBP terms enriched among genes downregulated in Msln^−/−^ aPFs include pathways related to ECM organization, immune signaling, and leukocyte chemotaxis. (*D*) Heatmap showing secreted proteins significantly downregulated in Msln^−/−^ aPFs, grouped by pathways identified in panel C. (*E*) GSEA of Hallmark gene sets comparing Msln^−/−^ with WT aPFs. Bar plot shows NES of significantly enriched gene sets (false discovery rate [FDR] < 0.25); representative enrichment plots are shown for EMT and IFN-γ response. (*F*) Expression of Msln, Thy1, fibrogenic genes, or inflammatory genes was analyzed by qRT-PCR in WT aPFs, Msln^−/−^ aPFs and Thy1^−/−^ aPFs. *Broken Y-axis* is used in Msln, Thy1, and Lgals1 to improve visualization of differences. Data are presented as mean ± SD. Comparisons between 2 groups were analyzed using the Mann-Whitney *U* test. Categorical data were analyzed by Fisher's exact test. ∗*P* < .05, ∗∗*P* < .01, ∗∗∗*P* < .001, ∗∗∗∗*P* < .0001.

The gene expression profiles of WT and Msln^−/−^ aPFs were accessed by RNA-seq. Differential expression analysis identified 466 significant differently expressed genes (DEGs) (adjusted *P* < .05; |log_2_fold change [FC]| > .58), with roughly equal numbers of genes up (n = 237) and downregulated (n = 229). As anticipated, Msln^−/−^ aPFs lacked expression of Msln, and strongly downregulated expression of fibrogenic (Tgfbi, Col1a2, Col3a1, Col6a3, Acta2, MMp2/3, Tnc, Timp1, Dpt) and inflammatory (Tnfaip6, Cxcl9, immunity-regulating interferon gamma [IFNγ]-inducible GTPases Tgtp1/2, Ccl4, Stat1, IL-1β, Ccl7, Ccn1) genes (Figure 9*A*; Supplementary Table 1).

### Msln^−/−^ aPFs Upregulate Genes Critical for Development and Cell Communications

Gene Ontology Biological Process (GOBP) overrepresentation analysis of upregulated genes revealed enrichment for developmental signaling and apical/basal polarity programs (Figure 9*B*). The top enriched term, “odontogenesis,” was driven by genes such as Tbx1 (transcription factors), Wnt7b (regulator of embryonic development), Lama5 (a component of basal membranes), and Nfic (transcription factor), which functions are broadly activated in mesenchymal development. Enrichment of genes within “establishment or maintenance of bipolar and apical/basal cell polarity” pathways was linked to upregulation of Wnt11 (plays a crucial role in early embryonic development, regulates MMPs), Scrib (scaffold protein that regulates cell motility), Ptk7 (regulates Wnt signaling), Crb2 (acts as adhesion molecule, regulates interaction of fibroblasts with epithelial cells), Camsap3 (regulates microtubule cytoskeleton organization), and Ezr (a part of ezrin/radixin/moesin family, regulates cell adhesion and motility) (Figure 9*B*; Supplementary Tables 2 and 3).

Importantly, genes downregulated in Msln^−/−^ aPFs were enriched for pathways associated with fibroblast activation and inflammation, including extracellular matrix (ECM) organization, leukocyte chemotaxis, and interferon signaling (Figure 9*C*), indicating reduced fibrogenic activity in Msln^−/−^ aPFs. To further characterize this shift in the gene expression profiles between WT and Msln^−/−^ aPFs, we examined expression of secreted proteins from these downregulated pathways and observed reduced expression of ECM regulators (Timp1, Tgfbi, Col1a2) and inflammatory mediators (Cxcl9, Tnfrsf9, Ccl7) in Msln^−/−^ aPFs (Figure 9*D*). Our data indicate that Msln^−/−^ aPFs exhibit a defect in activation and chemokine secretion.

### Msln^−/−^ aPFs Upregulated Genes That Inhibit Profibrogenic Wnt Signaling

To assess whether these changes reflected broader transcriptional changes, we performed Hallmark gene set enrichment analysis (GSEA). This analysis confirmed coordinated downregulation of immune and interferon signaling pathways, as well as epithelial-mesenchymal transition (EMT), further supporting a less activated phenotype of Msln^−/−^ aPFs (Figure 9*E*; Supplementary Table 4). Although the GO term reflects a tissue-specific annotation, the gene set pointed to Wnt-driven developmental programs, consistent with enrichment of the Wnt/β-catenin Hallmark pathway. Gene set analysis revealed that responses to estrogen and β-catenin were mostly suppressed due to the upregulation of inhibitors of Wnt signaling, Wnt6 (regulates post-natal tissue homeostasis), Dll1 (ligand for Notch receptor, regulates cell-to-cell communications), Numb1 (regulates Notch signaling, tumor suppresser), and Nkd1 (negative regulator of Wnt signaling by binding to Dishevelled (Dvl) proteins), whereas EMT and IFNγ responses were strongly reduced (as shown by ranked list of metrics and position in the ranked list of genes). Moreover, Msln^−/−^ aPFs upregulated Ncor2 (Nuclear Receptor Corepressor 2 that acts as a transcriptional corepressor), Nbl1 (BMP antagonist that NBL1 acts by binding to BMPs and preventing them from interacting with their receptors, thus inhibiting BMP signaling), and Gjb3 (a gap junction protein that provides instructions for making a protein called connexin 31) and others.^21^

### Expression of Lagls1, Mmp2, Mmp3, and Dpt Was Decreased in Msln-Deficient PFs and Increased in Thy1-Deficient PFs

Among the secreted factors identified in Figure 9*D*, expression of Lgals1, Mmp2, Mmp3, Dpt, Cxcl9, Tgfbi, and Tnc was significantly reduced in Msln^−/−^ aPFs. To validate our findings, expression of selected genes was measured in WT, Msln^−/−^, and Thy1^−/−^ aPFs. Expression of Lgals1 (gallectin1, involved in cell adhesion and migration), Mmp2 and Mmp3 (mediate tumor metastasis, and cell surface receptor cleavage), and Dpt (dermatopontin, codes for a protein involved in ECM organization and cell interactions) was suppressed in Msln^−/−^ aPFs but upregulated in Thy1^−/−^ aPFs (vs WT aPFs) (Figure 9*F*), suggesting that these factors regulate the development of cholestatic fibrosis and HCC in Mdr2^−/−^ mice. Specifically, qRT-PCR analysis confirmed that Msln^−/−^ aPFs downregulate Lgals1, Dpt, MMP3, and MMP2 (but not Cxcl9), and upregulate Tnc, leading to suppression liver fibrosis and improvement of hepatocyte regeneration. The mechanism by which Msln stimulates fibrogenic activation of aPFs has been described,^8^ whereas the role of Msln^+^ aPFs in hepatocyte proliferation is novel and has not been studied. The potential role of Lgals1, Dpt, Tnc, MMP3, and MMP2 in hepatocyte proliferation was further assessed.

### MMP3 Suppresses Proliferation of Hepatocyte Growth Factor-Stimulated Primary Human Hepatocytes

**Figure 10:**
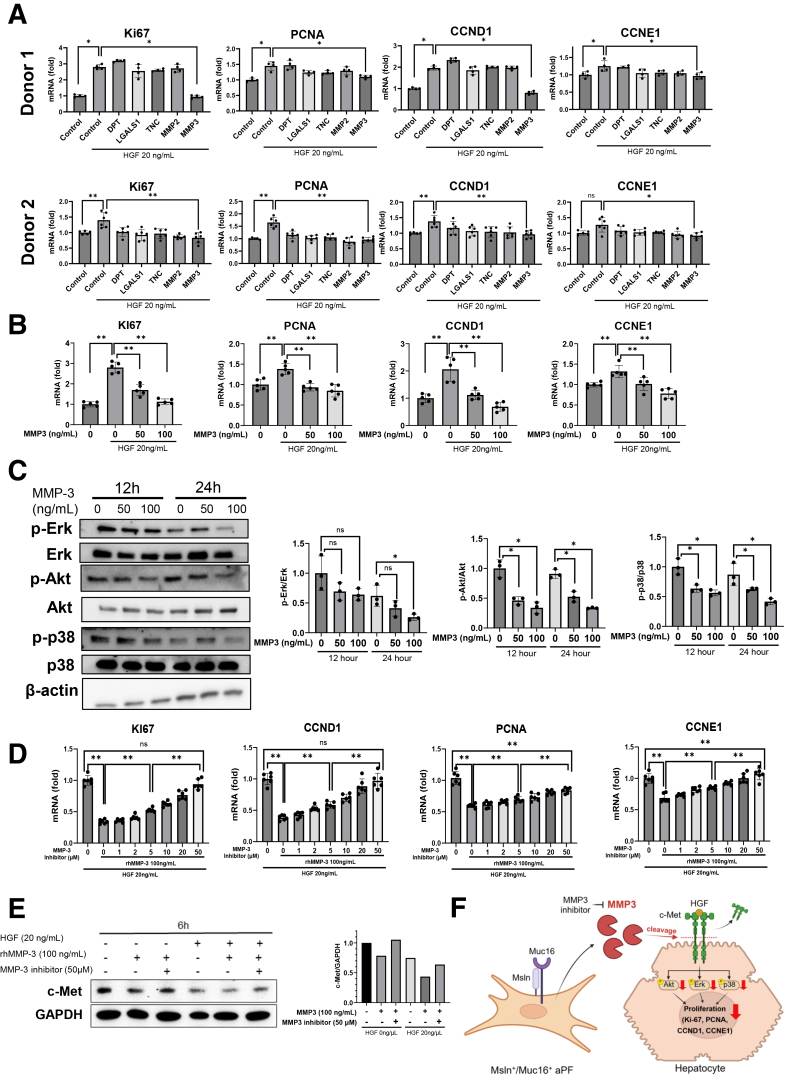
**MMP-3 inhibits hepatocyte proliferation by inducing Met degradation.** (*A*) Changes in the expression of proliferation markers in human hepatocytes treated with recombinant proteins and HGF. Recombinant DPT, LGALS1, TNC, MMP-2, or MMP-3 (100 ng/mL each) were administered to human primary hepatocytes from 2 different donors. (*B*) Evaluation of whether the HGF-induced increase in proliferation markers is altered by MMP-3 treatment in human hepatocytes. (*C*) Western blotting for c-Met downstream signaling molecules in human hepatocytes treated with HGF and MMP-3. Changes in protein expression were calculated as fold. (*D*) MMP-3 inhibitor restores expression of proliferation markers in MMP-3+HGF-stimulated human hepatocytes. (*E*) Expression of c-Met in human hepatocytes treated with HGF, MMP-3, and MMP-3 inhibitor. Data are presented as mean ± SD. Changes in protein expression were calculated as fold. The dot plot shows individual values. Comparisons between groups were analyzed using the Mann-Whitney *U* test. ∗*P* < .05, ∗∗*P* < .01, ∗∗∗*P* < .001, ∗∗∗∗*P* < .0001. (*F*) Graphical summary: aPF-derived MMP3 facilitates shedding of c-Met, thereby limiting hepatocyte proliferation.

The effect of select aPF-derived soluble factors on hepatocyte growth factor (HGF)-mediated hepatocyte proliferation was assessed in vitro in primary human hepatocytes (derived from 2 liver donors) (Figure 10*A*). Human hepatocytes (1.5 × 10^5^) were stimulated with ± HGF (20 ng/mL or vehicle) in the presence or absence of recombinant human dermatopontin (DPT; 100 ng/mL), galectin-1 (LGALS1; 100 ng/mL), tenascin C (TNC; 100 ng/mL), MMP2 (100 ng/mL), MMP3 (100 ng/mL) or vehicle for 24 hours. Only recombinant human MMP3 significantly suppressed proliferation of human hepatocytes (Figure 10*A*) in a dose-dependent manner, as shown by downregulation of Ki67, PCNA, CCND1, and CCNE1 (Figure 10*B*). This effect was associated with a strong downregulation of HGF-targets such as phospho(p)-Erk, p-Akt, p-p38 in HGF-stimulated hepatocytes in a time- and dose-dependent manner (Figure 10*C*). In line with these findings, MMP3-mediated suppression of HGF-induced hepatocyte proliferation was dose-dependently reversed by an MMP3 (Figure 10*D*), suggesting that aPF-derived MMP3 may affect hepatic HGFR(c-Met)-expression by facilitating proteolytic cleavage of c-Met and suppressing p-Erk/p-Akt signaling. In support, surface expression of c-Met protein was significantly reduced in MMP3+HGF-treated primary human hepatocytes MMP3 but restored in the presence of MMP3 inhibitor (Figure 10*E*). Our data identified a novel MMP3-dependent mechanism by which cholestasis-activated Msln^+^Muc16^+^ fibrogenic aPFs regulate hepatocyte proliferation. aPF-derived MMP3 facilitates shedding of c-Met, thereby limiting hepatocyte proliferation (Figure 10*F*).

## Discussion

Our study demonstrates that aPFs/mesothelial cells, activated by chronic cholestatic injury, play an important role in the pathogenesis of liver fibrosis and HCC (but not ICC) in aged female Mdr2^−/−^ mice. Although aPFs/mesothelial cells did not significantly contribute to the population of tumor-associated CAFs, fibrogenic aPFs appear to regulate hepatocyte growth, senescence, and malignant transformation of cholestasis-injured hepatocytes. Disruption of Msln-Muc16 signaling in aPFs/mesothelial cells in aged female Mdr2^−/−^ mice suppressed the development of liver cancer, especially HCC incidents, and this effect was attributed to reduced hepatocyte senescence, inflammation, and fibrosis. These findings outline a novel role of Msln-Muc16 signaling in aPFs/mesothelial cells in regulation of hepatocyte homeostasis and pathological responses.

More than 2% to 6% of patients with PSC/PBC and liver fibrosis develop HCC and ICC per year.^22^ The mechanism of hepatocarcinogenesis in cholestatic liver disease, specifically the role of aPFs, is not well-understood. Here we studied cholestasis-induced tumorigenesis in male and female Mdr2^−/−^ (Abcb4) mice,^23^ in which deletion of P-glycoprotein in the canalicular membrane of hepatocytes causes disruption of the bile duct tight junctions and basal membranes, causing bile leakage, periportal cholestatic fibrosis, and the development of cancer with age.^18^ The strain background and sex affect tumorigenesis in these mice, with females being more susceptible to cancer.^11^ Consistent with other studies, 16-month-old female Mdr2^−/−^ mice developed >4-fold more tumors than male Mdr2^−/−^ mice, and the tumors morphologically and phenotypically resembled hepatocellular adenomas or HCC (but not ICC), indicating that Mdr2^−/−^ mice serve as a useful tool to investigate the mechanism of HCC progression in cholestatic liver disease.

Using Mdr2^−/−^ mice, we evaluated the contribution of aPFs/mesenchymal cells in cholestasis-induced HCC. aPFs/mesothelial cells were identified by coexpression of αSMA and Thy1, CD34, Thy1, Msln, and Muc 16, which distinguished them from αSMA^+^Desmin^+^ aHSCs.^7^ Msln-Muc16 signaling was implicated in regulation of TGFβ1-TGFβRI-Smad2/3-dependent fibrogenic activation and FGF-Act1-ERK-STAT3-dependent proliferation of aPFs.^8^^,^^24^ Meanwhile, Thy1 was shown to prevent Msln-Muc16-TGFβRI signaling by blocking TGFβRI in aPFs. Binding of Msln-Muc16 complex to Thy1 resulted in dissociation of Thy1 from TGFβRI enabling TGFβ1 signaling in aPFs/mesenchymal cells.^8^

Genetic deletion of Msln and Muc16 reduced cholestasis-induced fibrosis and HCC in aged female Mdr2^−/−^ mice, whereas aged female Md2r^−/−^Thy1^−/−^ mice exhibited a phenotype similar to that in Mdr2^−/−^ mice. We hypothesized that Msln^+^Muc16^+^ aPFs can contribute to HCC via several mechanisms: (1) As described for ovarian and pancreatic cancers,^25^ Msln-Muc16 expression can be induced in malignant hepatocytes and drive HCC proliferation (compared with normal hepatocytes that do not express Msln-Muc16).^16^ Similar to other reports,^26^ our study demonstrated that Mdr2^−/−^ HCC expressed neither of these markers. Therefore, we focused on the role of Msln-Muc16-Thy1 signaling in aPFs. (2) Msln^+^Muc16^+^ aPFs/mesothelial cells can give rise to the tumor-associated CAFs that are known to support HCC growth and proliferation. Unlike aHSCs,^19^ aPFs minimally contributed to HCC-associated CAFs. (3) Peritumoral Msln^+^Muc16^+^ aPFs/mesothelial cells can provide Collagen-rich environment that facilitates tumor growth.^27^^,^^28^ In support, deletion of Msln could suppress the development of liver fibrosis and cancer.^29^ (4) Finally, similar to aHSCs,^30^ aPFs/mesenchymal cells can regulate fibroproliferative responses in injured hepatocytes/cholangiocytes of aged Mdr2^−/−^ mice. The later concept is novel and the role of aPFs in regulation of hepatocyte functions has not been previously described.

Specifically, our study revealed that neither HCC nor adenomas expressed Msln or Muc16 in aged female Mdr2^−/−^ mice, ruling out a possibility that Msln-Muc16 expression drives malignization of Mdr2^−/−^ hepatocytes. When the contribution of aPFs/mesenchymal cells to myofibroblasts was assessed in young mice, aPFs served as a significant source of ECM in response to cholestatic injury, comprising up to 70% of total myofibroblasts (100%), especially at the onset of injury.^7^ Surprisingly, in aged female mice, aPFs/mesothelial cells contributed to only 50% of total myofibroblasts in Mdr2^−/−^ mice. Genetic deletion of Msln and Muc16 resulted in a dramatic decrease of aPFs and suppression of liver fibrosis in Mdr2^−/−^Msln^−/−^ and Mdr2^−/−^Muc16^−/−^ mice vs Mdr2^−/−^ mice, suggesting that, despite reduced numbers, aPFs can regulate important processes in neighboring hepatocytes and cholangiocytes and liver microenvironment. Specifically, activation-associated Wnt signaling, and expression of inflammatory and fibrogenic genes are strongly reduced in Msln^−/−^ aPFs.

Similar to young mice, deletion of Msln and Muc16 attenuated bile duct proliferation and hepatic inflammation in aged female Mdr2^−/−^Msln^−/−^ and Mdr2^−/−^Muc16^−/−^ mice vs Mdr2^−/−^ mice, and correlated with reduced liver injury, suggesting that aPFs mediate a crosstalk between damaged cholangiocytes and inflammatory myeloid cells. This phenomenon can be attributed to reduced proinflammatory responses in aPFs and cholangiocytes, or shift of Msln- and Muc16-knockout aPFs/mesothelial cell functions from fibrogenic to regulatory in the peritumoral microenvironment. In accord, genetic deletion of Msln or Muc16 strongly suppressed hepatocyte senescence in aged female Mdr2^−/−^Msln^−/−^ and Mdr2^−/−^Muc16^−/−^ mice. The development of senescence-associated secretory phenotype (SASP) in hepatocytes promotes hepatic carcinogenesis and directly contributes to HCC.^25^, ^26^, ^27^ Senescent hepatocytes themselves can undergo malignant transformation due to the damage in the DNA repair machinery. Senescent hepatocytes/cholangiocytes were implicated in activation of fibroblasts.^15^ Here, we demonstrate that cholestasis-activated aPFs/mesenchymal cells regulate ductular reaction and hepatocyte/cholangiocyte senescence.

Moreover, proliferation of nontumor hepatocytes was improved in Mdr2^−/−^Msln^−/−^ and Mdr2^−/−^Muc16^−/−^ mice. Specifically, aPFs serve as a significant source of MMPs.^31^ MMPs were implicated in regulation of hepatocyte proliferation via shedding of HGF-receptor c-Met expression.^31^, ^32^, ^33^ Here, we demonstrate that HGF-mediated proliferation of human hepatocytes was suppressed in the presence of human recombinant MMP3 but restored by MMP3 inhibitor. We propose that aPF-derived MMP3 cleaves hepatic c-Met and disrupts HGF-c-Met signaling and AKT, ERK, p38 activation in hepatocytes,^34^ representing one of the mechanisms by which aPFs regulate/suppress hepatocyte proliferation. The effect of MMP3 on hepatocyte proliferation was reversed in the presence of MMP3 inhibitor. We concluded that suppression of Msln-Muc16 signaling in aPFs may ameliorate cholestatic fibrosis. (Of note, other identified Msln target genes DPT, LGALS1, TNC, and MMP2, appear not to play a role in hepatocyte proliferation, but may regulate distinct important functions in aPFs).

Targeting Msln^+^Muc16^+^ aPFs/mesothelial cells could become a therapy in patients with cholestatic fibrosis. We and others have shown that administration of anti-Msln antibody (Ab) can attenuate the development of cholestatic fibrosis in mice,^8^ and suppress differentiation of Msln^+^ fibroblasts into CALFs during pancreatic cancer.^35^ Based on our findings showing that blockade of Msln in aPFs/mesothelial cells ameliorates epithelial cell senescence and cholestatic fibrosis, more radical therapy can be implicated. Thus, ablation of aPFs/mesothelial cells using conditional Msln-ER-Cre^DTA^ transgenic mice, which inducibly upregulated Diphtheria toxin α in Msln^+^ aPFs/mesothelial cells, successfully attenuated the development of cholestatic fibrosis in BDL mice.^8^ Moreover, similar results were achieved when human aPFs/mesothelial cells were ablated using immunotoxic-coupled anti-Msln Abs.^9^ Currently, several classes of Msln-immunotoxins and anti-Msln CART cells have been generated and are being tested in Clinical Trials in patients with cancer.^36^^,^^37^ Similar therapies can be used in patients with cholestatic liver injury to suppress the detrimental effect of aPFs/mesothelial cells on HCC, fibrosis, inflammation, and cellular senescence.

In conclusion, our study demonstrates that Msln-Muc16 signaling regulates vital functions of aPFs/mesothelial cells in the liver. aPFs/mesothelial cell functions may change with age in mice with chronic cholestatic injury. Here, we provide the evidence that aPFs/mesothelial cells may regulate the balance between hepatocyte senescence and regeneration in aged liver.

## Materials and Methods

### Mice

Mdr2^−/−^ mice^10^^,^^23^ were crossed with Msln^−/−^ mice,^38^ Muc16^−/−^ mice,^39^ Thy1^−/−^ mice,^40^ and Col-GFP mice.^20^ Aged male and female littermates (C57BL6, 16 months, n ≥ 11–22/group) were housed under specific pathogen-free conditions in a standard environment with a 12-hour light-dark cycle and fed a diet of normal chow ad libitum at the animal facilities at the University of California San Diego under protocol S07088, approved by the Institutional Animal Care and Use Committee.

### Immunohistochemistry

Formalin-fixed paraffin-embedded mouse liver sections were stained with H&E, anti-GPC3 Ab (1:200; Abcam; ab95363), anti-Sox9 Ab (1:200; Millipore Sigma; AB5535), anti-phospho-Stat3 Ab (1:100; Cell Signaling Technology; 9145), anti-αSMA Ab (1:200; Abcam; ab5694), anti-pan-CK Ab (1:200; Dako; Z0622), anti-F4/80 (1:200; eBioscience; 14-4801-88), anti-CD34 Ab (1:200; eBioscience; 14-0341-81), anti-CK19 Ab (1:200, Abcam, ab5694), anti-CD4 Ab (1:200, Invitrogen, 14-9766-80), anti-MPO Ab (1:200, Abcam, ab139748), anti-CD31 Ab (1:200, R&D, AF3628), senescence associated β Galactosidase staining (Abcam; 1b65351), anti-p21 Ab (1:200; Abcam; ab188224), anti-Ki67 Ab (1:200; GeneTex; GTX16667), followed by 3,3′-diaminobenzidine (DAB) staining (Vector Laboratories). For immunofluorescent staining, anti-Msln Ab (1:150, Abbiotec, 250519), anti-Muc16 Ab (1:200, Abiocode, R2334-3), anti-Rabbit, anti-CD34 Ab (1:200; eBioscience; 14-0341-81), Donkey anti-Rabbit IgG (H+L) Alexa Fluor 594 (1:200, Thermo Fisher Scientific; A-21207), and Donkey anti-Rat IgG (H+L) Alexa Fluor 594 (1:200, Thermo Fisher Scientific; A-21209) were used as antibodies. Images were taken using IX-71 (Olympus), BZ-X710 (Keyence), and BZ-X800 microscopes (Keyence). Positive area was calculated as percent (using ImageJ); representative images of >2 independent experiments are shown.

### Histopathology Analysis of Liver Tumors

Histological evaluation was performed by a pathologist in a double-blinded manner based on the following criteria. HCC is a primary neoplasm of the hepatocytic origin in the liver, which manifests as distinct nodules of atypical hepatocytes with varying degrees of nuclear atypia in the mouse liver. The lesional tissue has abnormal hepatic and trabecular architecture with loss of complete portal tracts. HCC typically shows increased vascularization with isolated arterioles and sinusoidal capillarization. HCC in mice is frequently positive for AFP. In contrast, cholangiocarcinoma is an adenocarcinoma of intrahepatic bile ducts with gland formation. ICC can have a ductal or tubular pattern with a variable-sized lumen with endothelium, which contain intracellular mucin. The ICC cells are usually small or medium-sized, cuboidal or columnar, and can be pleomorphic. The nuclei are smaller and usually less prominent than in HCC.^41^

### Cholesterol Measurement

Cholesterol measurement was performed using mouse serum and liver tissues. Lipids were extracted from livers and serum using the Bligh and Dyer method, followed by quantification of total cholesterol using the Total Cholesterol Assay Kit (Cell Biolabs, Inc.; STA-384), according to the manufacturer’s instructions.

### qRT-PCR

Total RNA was extracted from tumor and nontumor liver tissues using TRIzol and Purelink RNA Mini Kit (Life Technologies). qRT-PCR was performed using QuantStudio 5 (Life Technologies). The primer sequences are shown in Supplementary Table1. Expression levels were normalized to HPRT by using the ΔΔ CT method.

### Western Blotting Analysis

Nontumor liver tissues and human hepatocytes were analyzed by Western blot using anti-Msln Ab (1:100, IBL, 28127), anti-Muc16 Ab (1:500, Abiocode, R2334-3), anti-αSMA Ab (1:1000; Abcam; ab5694), anti-βactin Ab (1:10000; Millipore Sigma; A5441), anti-phospho-Akt (1:1000, Cell Signaling Technology; #4060), anti-Akt (1:1000, Cell Signaling Technology; #4691), phospho-p38 MAPK (Thr180/Tyr182) (3D7) Rabbit mAb (1:1000, Cell Signaling Technology; #9215), p38 MAPK antibody (1:1000, Cell Signaling Technology; #9212), phospho-p44/42 MAPK (Erk1/2) antibody (1:1000, Cell Signaling Technology; #9101), p44/42 MAPK (Erk1/2) antibody (1:1000, Cell Signaling Technology; #4695), c-MET antibody (1:2000, Cell Signaling Technology; #3127), and GAPDH antibody (1:5000, Invitrogen; PA1-987).

### Partial Hepatectomy

WT and Msln^−/−^ mice (C57BL6, n > 4 female mice, 10 weeks old) were subjected to 70% partial hepatectomy, and livers were analyzed 3 days later.^42^

### Isolation and Culturing of Mouse aPFs and Hepatocytes

Primary aPFs were isolated from BDL-injured (5 days; n = 3/group) WT and Msln^−/−^ mice, sort purified for expression of Col-GFP and Thy-1 markers, and analyzed by RNA-seq^9^ or immortalized and used for the in vitro experiments.^8^ Nonstimulated WT and Msln^−/−^ aPFs were cultured (Dulbecco’s Modified Eagle Medium [DMEM] high glucose, fetal bovine serum [FBS] 10%) for 48 hours; the supernatant was collected and used to stimulate freshly isolated primary mouse hepatocytes for 48 hours ±HGF (40 ng/mL, R&D Systems, 2207-HG-025). Hepatocyte proliferation was evaluated by qRT-PCR.

### RNA-seq Analysis of WT and Msln^−/−^ aPFs

Gene-level expression was quantified using RSEM. Differential expression analysis was conducted using DESeq2 in R (v4.3.3), comparing Msln^−/−^ vs WT aPFs. Genes with an adjusted *P* value < .05 and absolute log_2_FC > 0.58 (1.5-FC) were considered significantly differentially expressed. Significant DEGs were subjected to GOBP overrepresentation analysis using clusterProfiler (v4.10.0). Hallmark GSEA was performed using the clusterProfiler and msigdbr (v24.1.0) packages. For GSEA, genes were ranked using the Wald statistic (“stat” column) from DESeq2 output. Gene sets with a GSEA q-value < .05 were considered significant. Volcano plots and normalized enrichment score (NES) bar plots were generated using ggplot2 (v3.5.1) with ggbreak (v0.1.5). Heatmaps of secreted proteins from downregulated pathways were constructed using ComplexHeatmap (v2.18.0) on log_2_-transformed transcripts per million (TPM) values. Only genes encoding secreted proteins, defined based on annotation, were included in the heatmap.

### In Vitro Studies in Human Hepatocytes

Human primary hepatocytes were isolated from 2 deidentified healthy donor livers (IRB 171883XX) which were obtained via Lifesharing OPO.^43^ Human hepatocytes (1.5 × 10^5^) were stimulated with recombinant DPT, LGALS1, TNC, MMP2, or MMP3 (100 ng/mL each) ± HGF (20 ng/mL); proliferation of human hepatocytes was evaluated 48 hours later. Recombinant human DPT (R&D, 4629-DP-050), LGALS1 (R&D, 1152-GA-050-CF), TNC (R&D, 3358-TC-050), MMP2 (R&D, 902-MP-010), MMP3 (R&D, 513-MP-010), and HGF (20 ng/mL, R&D, 294-HGN-025/CF) were used. To test the specificity of MMP3 effects, human hepatocytes were treated with HGF (20 ng/mL) ± recombinant MMP3 (100 ng/mL) ± MMP3 inhibitor (0 to 50 μM, Millipore SIGMA, 444218) for 6 hours (expression of c-MET was analyzed by Western blotting) or 48 hours (analyzed by qRT-PCR).

### Statistical Analysis

Data are presented as mean ± standard deviation (SD). Comparisons between 2 groups were analyzed using the Mann-Whitney *U* test. Categorical data were analyzed by Fisher’s exact test. The analyses were performed by Graph-Pad Prism software version 9.4.1 (GraphPad) and Image J Fiji.

## Supplementary Material

Supplementary Material

Extended PDF
