# Supplementary material for: Mesothelin/Mucin 16 Signaling in Activated Portal Fibroblasts Drives the Development of Cholestatic Fibrosis and Hepatocellular Carcinoma in Aged Female Multidrug Resistance Protein 2 Knockout Mice
Source: Cell Mol Gastroenterol Hepatol. 2026 Apr 11;20(8):101785. doi: 10.1016/j.jcmgh.2026.101785 (PMC13266261; doi:10.1016/j.jcmgh.2026.101785)

Figure2E Msln

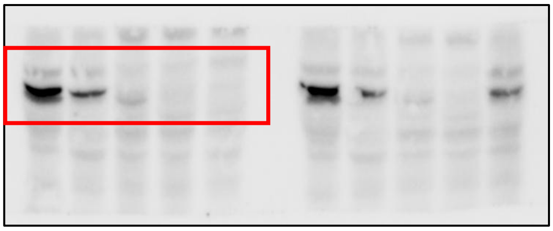

Figure2E β-actin

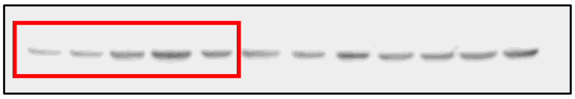

Figure2E Muc16

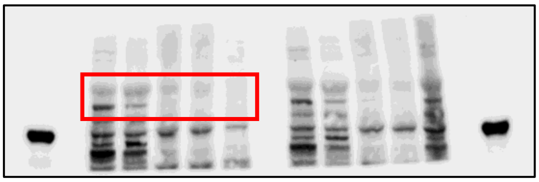

Figure2E β-actin

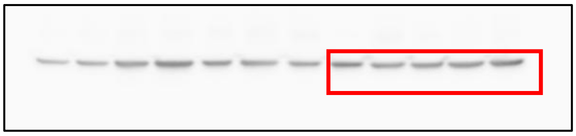

Figure4C αSMA

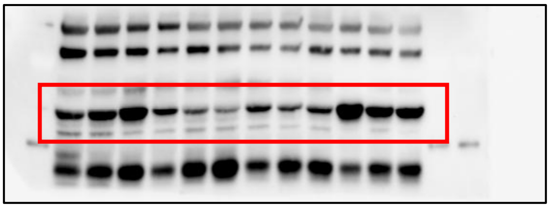

Figure4C β-actin

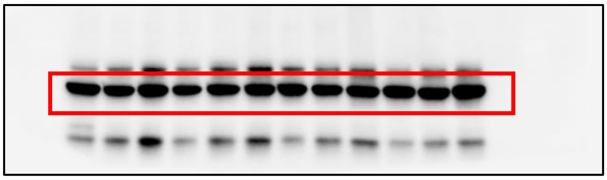

Figure8C p-Akt

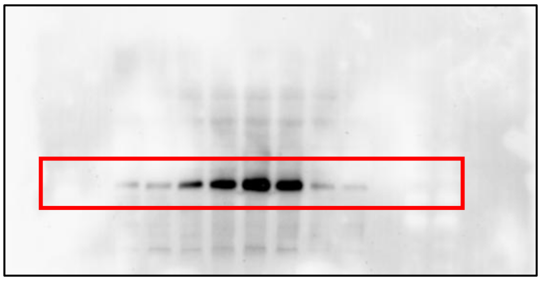

Figure8C Akt

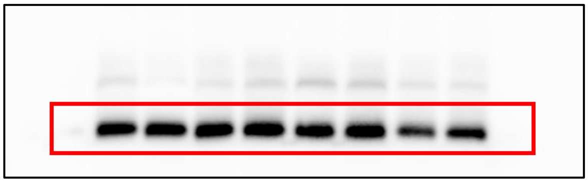

Figure8C p-p38

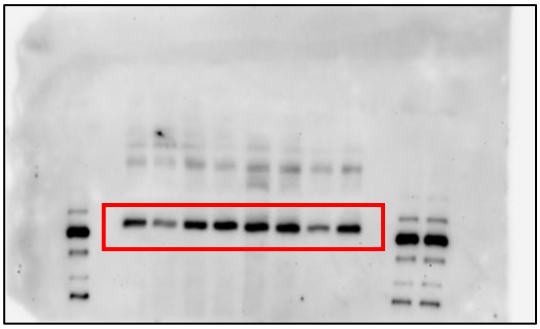

Figure8C p38

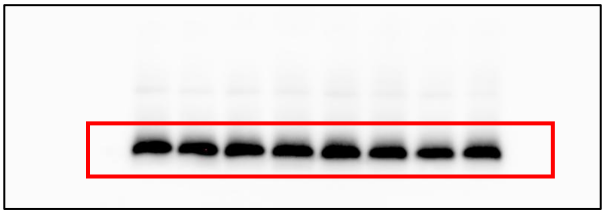

Figure8C β-actin

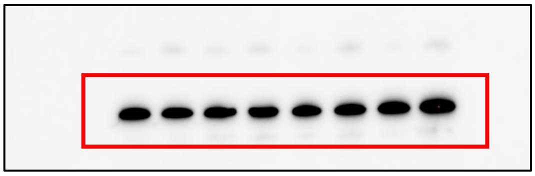

Figure8F p-Akt

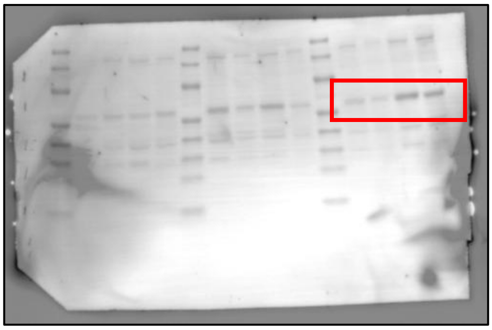

Figure8F Akt

Figure8F p-p38

Figure8F p38

Figure8F β-actin

Figure10D p-Erk

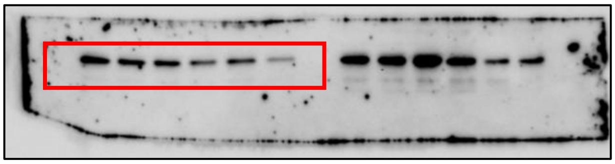

Figure10D Erk

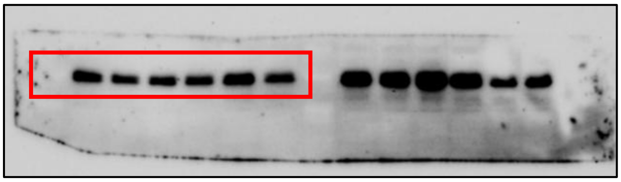

Figure10D p-Akt

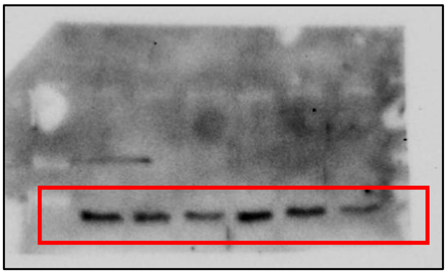

Figure10D Akt

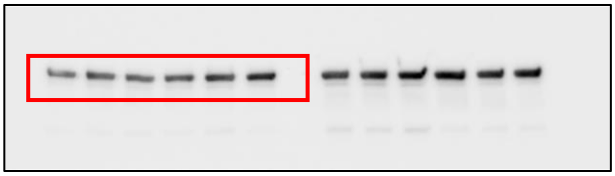

Figure10D p-p38

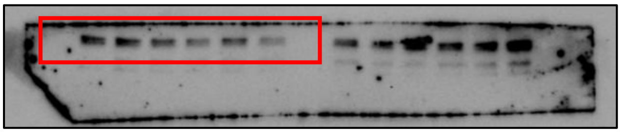

Figure10D p38

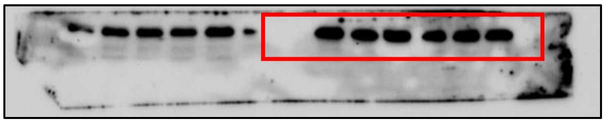

Figure10D β-actin

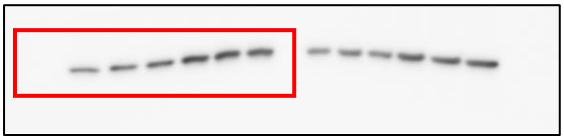

Figure10E GAPDH

Figure10E c-Met

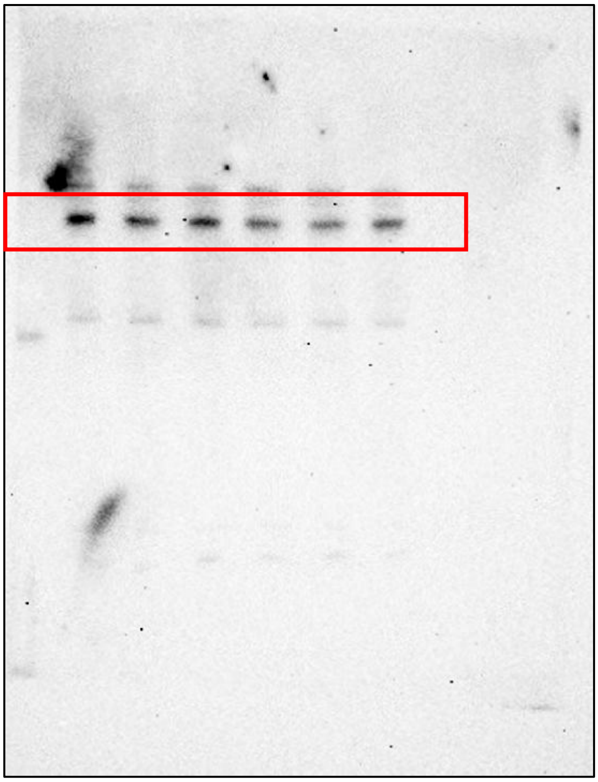

Figure10E GAPDH

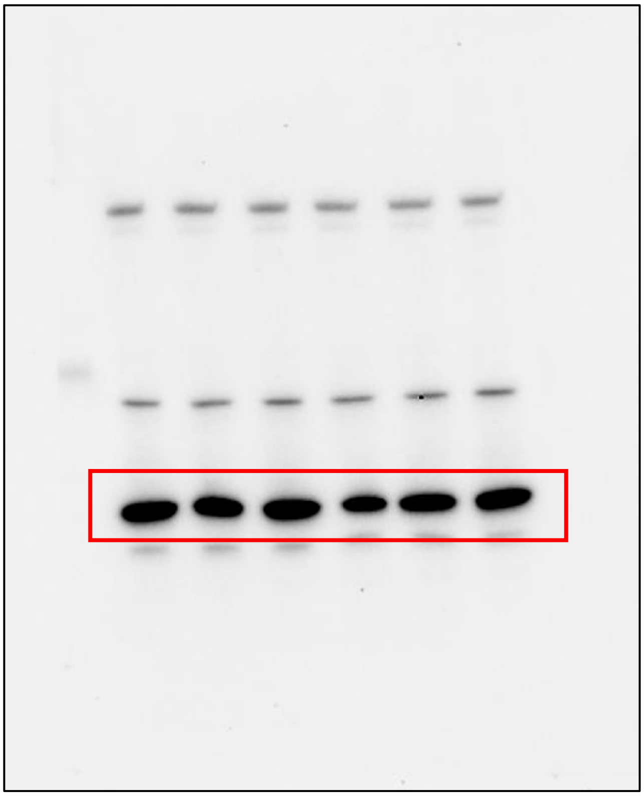

Supplement: Supplementary Material [file mmc5.pdf]
